# Impacts of El Niño Southern Oscillation on the dengue transmission dynamics in the Metropolitan Region of Recife, Brazil

**DOI:** 10.1590/0037-8682-0671-2021

**Published:** 2022-06-06

**Authors:** Henrique dos Santos Ferreira, Ranyére Silva Nóbrega, Pedro Vinícius da Silva Brito, Jéssica Pires Farias, Jaime Henrique Amorim, Elvis Bergue Mariz Moreira, Érick Carvalho Mendez, Wilson Barros Luiz

**Affiliations:** 1 Universidade Federal de Pernambuco, Programa de Pós-Graduação em Geografia, Recife, PE, Brasil.; 2 Universidade Federal de Campina Grande, Unidade Acadêmica de Geografia, Campina Grande, PB, Brasil.; 3 Instituto Nacional de Pesquisas Espaciais, Programa de Pós-Graduação em Computação Aplicada, São José dos Campos, SP, Brasil.; 4 Universidade Federal do Oeste da Bahia, Programa Multicêntrico de Pós-Graduação em Bioquímica e Biologia Molecular, Barreias, BA, Brasil.; 5 Universidade Estadual de Santa Cruz, Programa de Pós-Graduação em Biologia e Biotecnologia de Microrganismos, Ilhéus, BA, Brasil.; 6 Universidade Federal do Oeste da Bahia, Centro das Humanidades, Barreiras, BA, Brasil.

**Keywords:** Time-series, Climate changes, El Niño. Wavelets

## Abstract

**Background:**

This research addresses two questions: (1) how El Niño Southern Oscillation (ENSO) affects climate variability and how it influences dengue transmission in the Metropolitan Region of Recife (MRR), and (2) whether the epidemic in MRR municipalities has any connection and synchronicity.

**Methods:**

Wavelet analysis and cross-correlation were applied to characterize seasonality, multiyear cycles, and relative delays between the series. This study was developed into two distinct periods. Initially, we performed periodic dengue incidence and intercity epidemic synchronism analyses from 2001 to 2017. We then defined the period from 2001 to 2016 to analyze the periodicity of climatic variables and their coherence with dengue incidence.

**Results:**

Our results showed systematic cycles of 3-4 years with a recent shortening trend of 2-3 years. Climatic variability, such as positive anomalous temperatures and reduced rainfall due to changes in sea surface temperature (SST), is partially linked to the changing epidemiology of the disease, as this condition provides suitable environments for the *Aedes aegypti* lifecycle.

**Conclusion:**

ENSO may have influenced the dengue temporal patterns in the MRR, transiently reducing its main way of multiyear variability (3-4 years) to 2-3 years. Furthermore, when the epidemic coincided with El Niño years, it spread regionally and was highly synchronized.

## INTRODUCTION

Dengue is an arboviral disease, mainly vectored by *Aedes aegypti* (primary vector) and *Aedes albopictus* (secondary vector), caused by four distinct virus serotypes of the Flaviviridae family, genus Flavivirus (dengue virus (DENV)-1, DENV-2, DENV-3, and DENV-4)^1^. Infection with one of these serotypes induces lifelong immunity against the serotype and temporary immunity against the others^2^. However, secondary infections by distinct serotypes increase the risk of severe dengue^3^ ^,^ ^4^, particularly in susceptible populations^5^ ^,^ ^6^. This group is highly heterogeneous, composed of children under 10 years of age, cases of reinfection with DENV-2 serotypes, and adult females^7^ ^-^ ^9^.

Currently, dengue mainly affects poor and vulnerable populations^10^. Endemic in more than 128 countries^11^ ^,^ ^12^ is a human arbovirus of quick spread^13^ ^,^ ^14^. Global estimates have shown significant challenges in the control and prevention of epidemics. Between 1990 and 2013, there were 10,000 deaths and 100 million cases of symptomatic dengue infections per year^15^. Approximately 4 billion people worldwide are at risk of being infected with dengue^16^, mainly in South America, Southeast Asia, and Central Africa^17^. Global warming may contribute to the geographic expansion of this disease into new areas^17^ ^-^ ^20^, including the Southeastern USA, high altitudes of Central Mexico, Northern Argentina, Australian hinterland regions, the Eastern coast of China and Japan, Southern Africa, and the Sahel region of West Africa. If this occurs, an estimated 60% of the global population will be at risk of dengue infection by 2080^17^.

Climate variables are important regulators of *Ae. Aegypti* lifecycle, which affects the viral replication rate inside the vector, the mortality rate of affected populations, and the behavior of mosquitoes^21^ ^-^ ^23^. Several studies have revealed the relationships between climate variables and dengue transmission, with many showing the specific climatic conditions of each environment, such as the relationship between rainfall variability and local temperature with optimal viral transmission^22^ ^,^ ^24^ ^,^ ^25^. However, some uncertainties remain, such as the intensity and frequency of climate impacts associated with the El Niño Southern Oscillation (ENSO)^26^.

Other conditioning factors are also responsible for the establishment of the dengue epidemic^27^. For example, human mobility, population density, economy, sanitation conditions, and health assistance affect its real distribution within climate variables^28^ ^,^ ^29^. Thus, this study aims to answer two questions: (1) how ENSO affects climatic conditions and how it influences dengue dynamic transmission in the metropolitan region of Recife (MRR) and (2) whether the epidemic in MRR municipalities has any connection and synchronicity.

## METHODS

### Study Area

**FIGURE 1:**
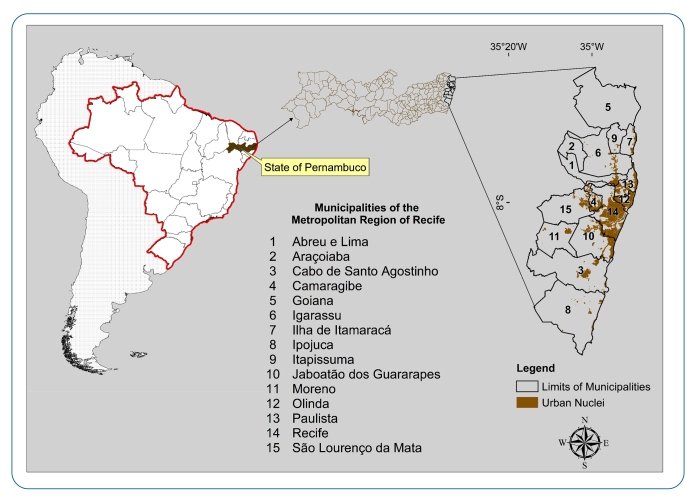
Location of municipalities that compose the Metropolitan Region of Recife, Pernambuco, Brazil.

The MRR is located in the “Zona da Mata” region of the state of Pernambuco (Figure 1). It has 15 municipalities, approximately 3,216,262 km², 4,054,866 inhabitants, and a population density of 1,260.74 inhabitants/km², according to the Brazilian Institute of Geography and Statistics (IBGE). Regarding the local climate, from 1980 to 2016, the MRR presented a mean annual accumulated precipitation of 858 mm (minimum) and 2,539 mm (maximum), with a mean annual value of approximately 1,625 mm. The rainy season occurs during the autumn/winter period. The monthly mean temperature varied from 28.49 °C to 30.88 °C, while the mean monthly minimum and maximum temperatures oscillated from 20.66 °C to 23.34 °C and from 26.68 °C to 32.55 °C, respectively.

### Data - dengue and climatic variables

We defined two distinct periods for the analysis: from 2001 to 2017 (analysis of the periodicity of the dengue incidence and intercity epidemic synchronism), and from 2001 to 2016 (due to availability of climatic data) to analyze the periodicity of climatic variables and their coherence with the incidence of dengue. The incidence rates per 100,000 people were calculated by municipality using the number of cases confirmed in the laboratory and by clinical-epidemiological criteria obtained through the Notifiable Diseases Information System (NDIS): http://www2. datasus. gov. br/DATASUS/index. php?area=0203&id=29878153. The population census of 2010 and other years were obtained from the IBGE. Monthly climate data were obtained from databases with fill gaps^30^, including accumulated precipitation and the maximum and minimum temperatures. For municipalities without climate stations, the data from the closest stations were used. In the regional analysis, the incidence rates and climatic variables were given by the mean of the municipalities.

ENSO is an ocean-atmosphere climatic system that induces cyclical changes in climatic variables in several regions of the globe^31^. El Niño (positive phase of ENSO) occurs, on average, every 3-7 years^32^ ^,^ ^33^, with episodes typically lasting 9-12 months^33^, and is characterized by sea surface temperature (SST) above the mean in region 3.4 of the equatorial Pacific^33^. ENSO is the Earth’s strongest interannual climate cycle and is the main cause of climatic variability in Northeastern South America^32^ ^,^ ^34^. El Niño is classified into three categories: weak (0.5 to 0.9 SST anomaly), moderate (1.0 to 1.4 SST anomaly), and strong (anomaly > 1.5 SST anomaly)^33^. SST was used here as a proxy for El Niño and not El Niño to analyze how ENSO influenced climatic variables and dengue incidence rates. SST data were obtained from the National Oceanic and Atmospheric Administration/Climate Prediction Center NOAA/CPC: https://www.cpc.ncep.noaa.gov/data/indices/.

### Periodicity - dengue, ENSO, and climatic variables

Wavelet analysis was used to investigate changes in the rhythmic pattern over time for climatic time series and dengue incidence rates. This technique is efficient for this approach because wavelet analysis is a spectral-specialized method that allows the analysis of the frequency of events at different scales of temporal variability^35^. In a complex series, statistical properties change over time, making them highly noisy and unsuitable for analysis using classic methods^36^. Before analysis, each monthly observation in the all-time series was transformed into square roots and standardized by the mean and long-term standard deviation. The Morlet wavelet, which is frequently used in the analysis of natural signals, consists of 1:

ψt=π-14 eiω0te-t22 (1)

where ω_0_ denotes the dimensionless frequency. Here, we used ω_0_ = 6 to satisfy the admissibility condition.

A wavelet function must be able to decompose and represent another function at distinct frequency and time scales. Therefore, wavelets are a family of functions derived from a single function, denoted by the mother wavelet (equation 2):

$$\psi_{a,\tau }(t)=\frac{1}{\sqrt{a}}\psi \left(\frac{t-\tau }{a}\right),a\in R^{+}eb\in R$$ (2)

where τ indicates the distance that function $\psi_{a,\;\tau }(t)$ is translated on the t axis (position of time); *a*, denoted as a scale parameter, represents function dilation if *a* > 1 or contraction if *a* < 1 in the signal; $\frac{1}{\sqrt{a}}$ as a normalization factor, ensures that the energy of $\psi_{a,\;\tau }(t)$ is independent of *a* and τ parameters.

The mother wavelet is calculated using parameters*a* = 1 and τ = 0, in which it does not influence the function, and for any other a and τ values, daughter wavelets were obtained. The decomposition with the wavelet analysis function is called the wavelet transform of time series *x*(*t*) for a given mother wavelet, in this case, the Morlet wavelet. The wavelet transform decomposes a function defined in the domain into another function defined in the time and frequency domains, as determined by

$$W_{x\left(a,\tau \right)}=\;\frac{1}{\sqrt{a}}\int_{-\infty }^{\infty }x\left(t\right)\psi *\left(\frac{t-\tau }{a}\right)dt=\int_{-\infty }^{\infty }x\left(t\right){\psi *}_{a,\;\tau }\left(t\right)dt$$ (3)

where * denotes the complex conjugate and ^*W*^ *x*(*a*,τ) are the wavelet coefficients. The purpose of wavelet transform is to be proportional and exact to the scale. Therefore, it studies the signal at different scales and moves by analyzing each part of the signal until all structures that are a part of the signal are found. Using the wavelet, it is possible to estimate the wavelet power spectrum (WPS) (equation 4), which is the decomposition of the variance between the scale parameter and the time location (t).

$$S_{x}\left(f,t\right)={\left|W_{x}(f,t)\right|}^{2}$$ (4)

### Intercity synchronicity and the influence of climatic factors on dengue transmission - wavelet coherence (WC) and phase analysis

The wavelet coherence (WC) is a mathematical technique of direct measurement between spectra of two-time series^37^, which can detect whether two signals simultaneously oscillate on specific time scales and frequencies and is applied in this study to quantify the statistical associations between two nonstationary time series. In addition, phase differences were measured to characterize the time function between the time series and reveal synchronous or asynchronous patterns over time^38^. WC and phase differences were determined for municipalities that were not contiguous and contiguous to Recife. Furthermore, other tests with similar criteria were applied to assess the intercity synchronism of two other sets of municipalities: the first in the North and the second in the South of Recife. The intention was to investigate outbreak patterns that could stem from underlying factors, such as human mobility. WC was calculated using equation 5:

$$R_{x,y}(f,\tau )=\frac{{\left|s\left(W_{x,y}(f,\tau )\right)\right|}^{2}}{{\left|s\left(W_{x}(f,\tau )\right)\right|}^{2}{\left|s\left(W_{y}(f,\tau )\right)\right|}^{2}}$$ (5)

where $W_{xy}(f,\tau )=W_{x}(f,\tau )W_{y}*(f,\tau )$ express the cross*-*wavelet transform, $W_{x}\left(f,\tau \right)$ and $W_{y}\left(f,\tau \right)$ represent the wavelet transform of series *x*(τ) and *y*(τ) , respectively, and s is a smoothing operator, in time and frequency .

Coherence allows the estimation of how two nonstationary time series *x*(τ) and *y*(τ) are related at different frequencies over time. $R_{x,y}\left(f,\tau \right)$ varies from 0 to 1 and indicate a strong or weak relationship for low or high values, respectively, and have a perfect linear relationship when $R_{x,y}\left(f,\tau \right)=1$ between two signals at a given frequency and time.

Moreover, it was possible to calculate the phase difference with wavelet transforms and instantaneous time delay ∆Τ(τ) between the two temporal series *x*(τ) and *y*(τ); they are mathematically expressed in equations (6) and (7), respectively:

$$R_{x,y}(f,\tau )=\frac{{\left|s\left(W_{x,y}(f,\tau )\right)\right|}^{2}}{{\left|s\left(W_{x}(f,\tau )\right)\right|}^{2}{\left|s\left(W_{y}(f,\tau )\right)\right|}^{2}}$$ (6)

where *I* represents the imaginary part and *R*, the real part.

$$\Delta T(\tau )=\frac{\phi_{x,y(f,\tau )}}{2\pi F(\tau )}$$ (7)

Wavelet analysis was performed using Matlab R2020a (The Math- Works, Inc., Natick, Massachusetts, United States), properly licensed for student use, while toolboxes used in the analysis were acquired from Cazelles et al. (2005)^39^.

### Quantifying the association between time series

Cross-correlation functions (CCF) were used to quantify how much time series were related due to a lag applied to one of them. Initially, each variable is decomposed to separate its components. An autoregressive integrated moving average (ARIMA) model was then adjusted for residuals, and statistical metrics were applied to validate the absence of autocorrelation and data stationarity. This method was obtained from time series analysis: Forecasting and Control^40^.

## RESULTS

### Interannual dengue variability in MRR

**FIGURE 2:**
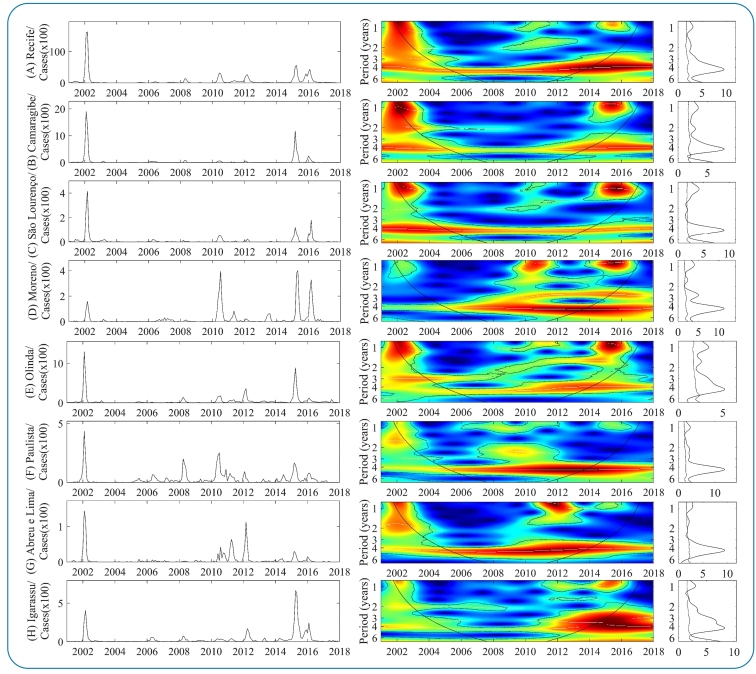
WPS of the monthly dengue incidence between 2001 and 2017 in the 15 MRR municipalities Left graph: time series of the number of cases by a municipality. Color codes indicate increasing intensity from blue to red. Broken black lines show statistically significant areas (threshold of 5% confidence interval). The panels on the right correspond to Spectrum Global (GS).

**FIGURE 3:**
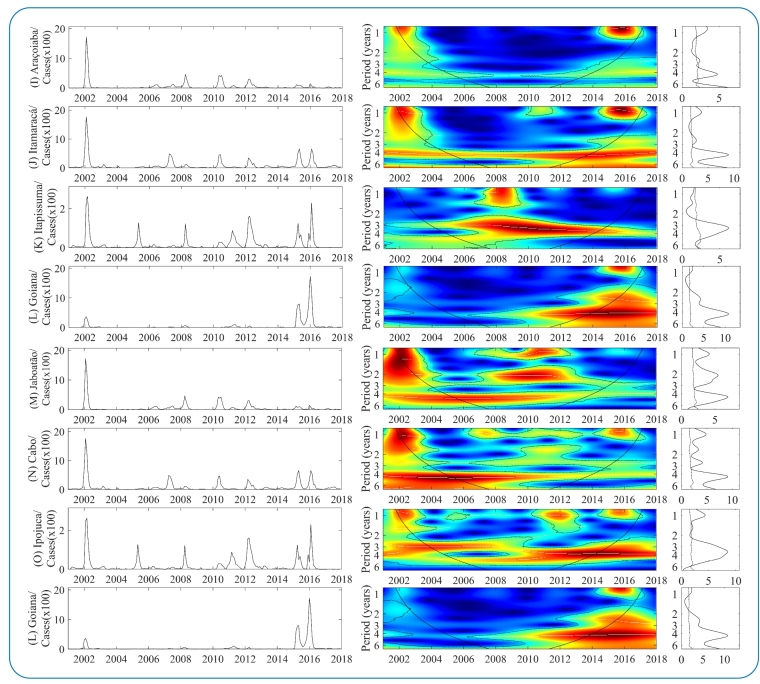
Color description, like the other parameters, is shown in Figure 2

Figures 2 and 3 show the results of the continuous wavelet transform (CWT) performed on the dengue incidence series in each MRR municipality. Generally, the dengue dynamics in municipalities showed statistically significant spectral regions in seasonal and multiannual frequencies, with continuous and transient temporal patterns over time, varying from low to high power. Regarding multiannual cycles, statistically significant medium and strong spectral regions are dominant and continuous throughout the period in the 3-4 years band of time series of 13 municipalities (Figures 2 [A-H]; Figures 3 [J-K and M-O]) suggest an intense return of epidemics every 3-4 years. However, after 2010, some municipalities started showing a trend of shortening epidemic cycles of the main multiannual variability cyclical mode from 3-4 years to 2-3 years. This change in the epidemiological pattern can be associated with intense factors that amplify epidemic cycles, such as the cross-protective immunity of the population group or climatic variability.

The most intense epidemic cycles occurred during three main periods (2002-2003, 2006-2012, and 2014-2017). In the first and third cycles, the epidemic spread across almost all MRR municipalities (Figure 2 and 3, graphs on the left). Regarding the seasonal module, three statistically significant regions, ranging from medium to high power, were computed: (2002-2003, 2006-2012, and 2014-2017). The first was strongly predominant in municipalities shown in Figures 2 (A*-*C), Figure 2 (E), and Figures 3 (J and M*-*O); the second exhibited medium spectra in Figure 2 (F), Figures 3 (M and N) (2006-2012), and Figures 2 (D and E) (2008-2012); and the last exhibited dominant strong spectra in Figures 2 (B*-*E), Figures 2 and 3 (H*-*J), and Figures 3 (L and N*-*O). In addition, there are trends in the recent increase in the magnitude of epidemics in most municipalities, mainly in Igarassu (Figure 2 [H]) and Goiana (Figure 3 [L]), where incidence rates have been the highest in recent years.

### Intercity dengue synchronicity

**FIGURE 4:**
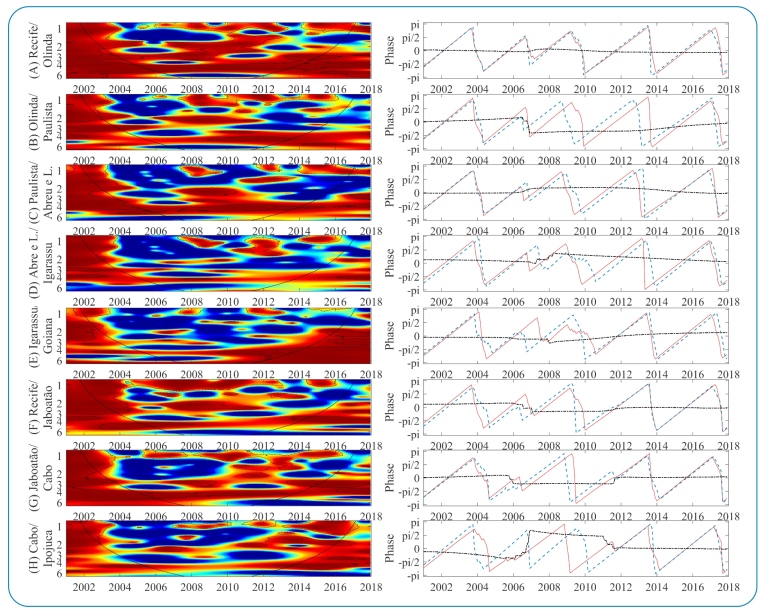
CSW of dengue incidence on the left, spectra ranging in intensity from blue (low coherence) to red (high coherence). Dashed lines identify periods whose coherence was statistically significant, and the solid black line delimits regions without border effects. On the right, the phase relationship in the 3-year band (red line: first municipality; blue line: second municipality; and black dotted line: phase difference between series).

**FIGURE 5:**
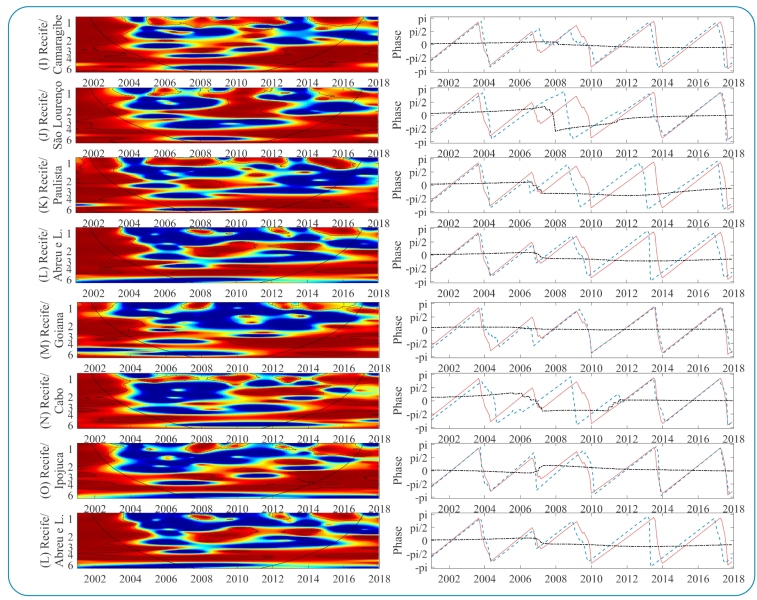
Color description, like the other parameters, is shown in Figure 4.

The cross-spectrum wavelet (CSW) of dengue epidemiological time series in MRR municipalities is shown in Figures 4 and 5, and on the right, the phase difference distribution of the two-time series in the 3-4 year band. The CSW determines the coherent oscillations in a specific frequency and period, while the phases reveal the time synchronization or time lag of one signal with respect to the other for the main multiannual variability mode (3-4 years). In general, the results showed high coherence between time series with different intensities and variations in frequencies and time intervals.

Intercity epidemic synchronism is prominent when municipalities share administrative boundaries. The CSW analysis revealed that the municipalities of Recife and Olinda had more evident coherence and timing (Figure 4 [A]), especially for the dominant variability mode in both series (3-4 years). A second periodic band (2-3 years) also showed coherence and synchronization during the two main periods (2001-2005 and 2006-2012). Oscillations in the 3-4 years band of the municipalities of Paulista and Abreu e Lima (Figure 4 [C]) were synchronized until 2015. Initially, oscillating in phase, after 2006, the dengue epidemic in Paulista preceded Abreu and Lima by approximately 1 month. Similarly, dengue in Paulista preceded dengue in Olinda (Figure 4 [B]) from 2008 to 2014 and Recife from 2008 to 2016. This suggests that the epicenter was Paulista, which progressively spread to surrounding municipalities. However, this pattern was transitory and limited to neighboring municipalities.

### Dengue, SST, rainfall, and temperature variability

**SUPPLEMENTARY FIGURE 1:**
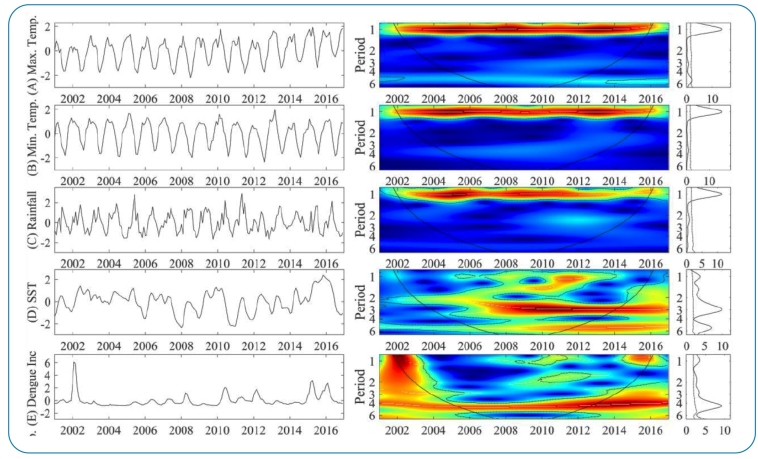
Maximum interannual temperature variability **(A)**, minimum temperature **(B)**, rainfall **(C)**, SST **(D)**, and dengue panels on the left correspond to time series standardized by the long-term mean and standard deviation, and on the right, wavelet power spectra ranging in intensity from dark blue (low-power) to red (high-power). The dashed black lines indicate that the wavelet power spectral regions were statistically significant.

The interannual maximum (A) and minimum temperature (B), precipitation (C), SST (D), and dengue (E) variabilities are shown in Supplementary Figure 1. The temperature and precipitation series showed continuous oscillations in the seasonal band, whereas SST and dengue had periodic high-power cycles in the multiannual frequencies of 2-3 years (2006-2016) and 3-4 years (throughout the period). Interannual changes in precipitation after 2011 were also detected when the high power was interrupted, and the medium spectrum started dominating the seasonal band, suggesting a reduction in the mean accumulated monthly rainfall compared with the previous period. This reduction is befitted with the SST positive anomaly observed from 2011 to 2016 (Figure 4 [C], graphics on the left). Furthermore, for the same period (2011-2016), a trend of increase in maximum temperatures were observed and a change in the epidemiological dengue incidence pattern in the regional scenario, with a trend of reduction in interepidemic periods.

### Climatic variables and dengue incidence in MRR

**SUPPLEMENTARY FIGURE 2:**
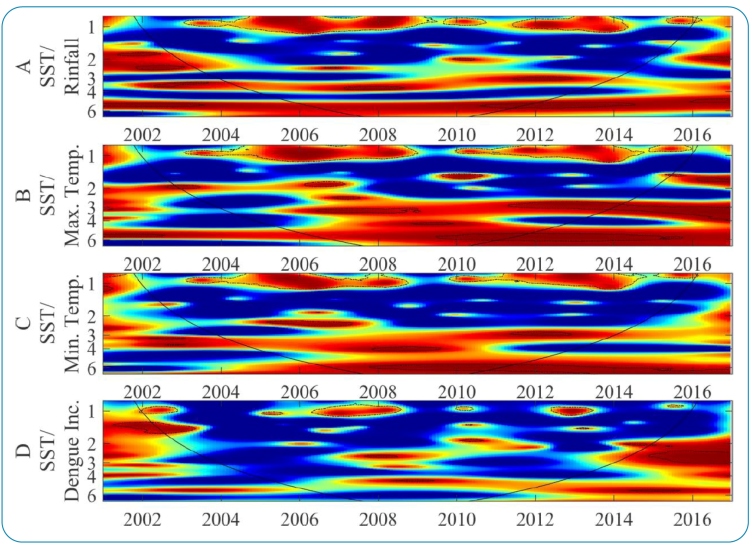
WC analysis between SST and rainfall **(A)**, SST and Maximum temperature **(B)**, SST, minimum temperature **(C)**, SST, and dengue **(D)**. The color description, similar to that of the other parameters, is shown in Figure 4.

Supplementary Figure 2 shows the coherence between SST and the climatic time series, SST, and dengue incidence. Highly coherent oscillations between SST and climatic variability in the seasonal band were observed. These oscillations are transitory in time and stand out in four main significant spectral regions (2001-2002, 2006-2011, 2012-2013, and 2015-2016). The same pattern was observed for seasonal variability between the SST and dengue. In the multiannual frequencies, periods of high coherence were detected between SST and maximum temperatures from 2011 to 2016 (2-3 years band) and in the 4-6 years band (2010-2016). Only two regions showed high coherence in dengue incidence in the multiannual mode. The first is the periodicity of 1-2 years (2001-2003), and the second is the periodicity of 2-3 years from 2014 to 2016. However, both regions are inside the influence cone, and some edge effects can influence this outcome. Therefore, new studies should cover the period prior to 2001 and posterior to 2016 to provide more consistent evidence on the role of climatic variability induced by El Niño over dengue dynamics in the MRR. This approach avoids a possible edge effect on the results.

### Statistical association between the climatic and dengue incidence time series

The CCF between (i) climatic variables and dengue, (ii) SST and dengue, and (iii) SST and climatic variables were calculated. Significant correlations between SST and dengue incidence (lag of 9 months and r = 0.23), SST and rainfall (lag of 7 months and r = -0.18), and SST and maximum temperature (lag of 2 months and r = 0.15) were observed. Furthermore, significant and positive correlations between rainfall and dengue incidence and significant and negative correlations between maximum temperature and dengue incidence were observed, suggesting that changes in precipitation could influence dengue dynamics.

## DISCUSSION

A trend of shortening interepidemic intervals from 3-4 years to 2-3 years was observed, a pattern similar to that observed for the dynamics of dengue transmission in Brazil in the last years^41^. Our findings indicate that climatic variability caused by ENSO (positive phase) is one of the factors associated with the recent epidemiological changes in dengue transmission in the MRR, which is consistent with the results obtained by Atique et al. (2016)^42^. The context of the decrease in accumulated rainfall and the increase in maximum temperature induced by positive SST anomalies may provide more adequate conditions for the dengue vector lifecycle. These results are in line with those of Vincenti-Gonzalez et al. (2018)^43^, who found that biennial and triennial dengue cycles related to El Niño were caused by climate variability.

After controlling for the effects of seasonal variability and autocorrelation in the time series, it had demonstrated that monthly cumulative precipitation anomalies had a 7-month lag regarding the changes in SST. Similarly, SST had positively correlated with maximum temperature with a 2-month lag and an average increase in dengue incidence with a 9-month lag. Furthermore, the main epidemics in the region coincided with two moderate El Niño 0 events (2002 and 2010) and one El Niño + 1 year (2015-2016 - strong El Niño). These results can be linked to long-term changes in atmospheric conditions, which have become potentially favorable to dengue outbreaks, as observed by Anyamba et al. (2019)^44^ during the strong 2015-2016 El Niño event, exceptionally favorable for vector-borne diseases, including dengue, and are in line with previous reports^29^ ^,^ ^39^.

Although these results indicate an increase in dengue incidence associated with a decrease in rainfall, it was also observed that the winter preceding an epidemic year showed strong predictive power for dengue, corroborating recently reported results^25^ ^,^ ^45^. This lag in the dengue response regarding rainfall may provide relevant information to intervention measures in the appropriate period, as reported by Stolerman et al. (2019)^25^, who detected critical climatic conditions until 9 months before the epidemic peak and highlighted that a large initial vector population combined with several reproduction cycles might lead to important outbreaks in the summer.

Our results add to the growing scope of evidence that suggests regional signals of the effects of ENSO on dengue transmission dynamics^29^ ^,^ ^46^ ^,^ ^47^ ^,^ ^48^ ^,^ ^49^. However, there are uncertainties regarding the impact of ENSO on MRR, which has future intensification scenarios^34^. These scenarios show a decreasing tendency in rainfall and an increasing tendency in local temperatures. If confirmed later, the dengue epidemiological situation in the region will worsen. In addition to climatic determinants, the incidence of dengue depends on immunological factors^50^ ^,^ ^51^. In addition to the permanent immune response after infection by one strain, the cross-protection given to other dengue virus serotypes may also influence the length of interepidemic periods^50^ ^,^ ^41^. This cross-protection can last from 2 to 3 months^52^ and confer temporary immunity to population groups recently exposed to major epidemics. As El Niño showed larger predictive power for dengue, we believe that this would provide information for a control intervention that has been rarely explored by health authorities. This approach goes against the practice of dengue epidemiological control in Brazil, where local interventions are limited to spring and summer.

Strong intercity synchronicity was observed between the dengue time series, especially during the epidemic connected to the ENSO (2002, 2010, and 2015/2016). A similar dynamic was reported by Cazzeles et al. (2005)^39^ in Thailand. In our results, synchronicity was clearer among municipalities that shared administrative boundaries or were in the context of the urban conurbation, as in Recife and Olinda’s case. It was notably consistent with the dengue dynamics in the state capital, even in more distant municipalities such as Goiana. Therefore, the state capital is a possible source of origin or an articulator of dengue epidemics in the MRR when atmospheric conditions are favorable. Similar results have been reported in Southern Taiwan, where the epidemic has spread from large cities to smaller cities^39^ ^,^ ^53^. Associated with atmospheric conditions, adjacent processes such as the spatial hierarchy of population structure^54^ and human mobility^55^ ^-^ ^58^ could play an important role in the spread of the epidemic. When this synchrony is regionally strong, it could have important consequences: a health system collapse^58^ and an increase in disability-adjusted lost life Years-DALY^59^.

 Synchronous and periodic epidemics in different population groups have other important implications regarding epidemiological control. For example, epidemic eradication becomes easier when synchronized, either through vaccination or control and prevention actions. In contrast, prevention and control challenges are more expensive when asynchronous because of the risk of reintroduction in places where it has been eradicated or controlled^60^, especially in endemic locations such as metropolitan regions. However, periodicity can be related to inefficient control and prevention actions^41^. Therefore, determining epidemic synchronism and regularity at the metropolitan scale shows how the disease evolves and can be connected between municipalities. However, it is imperative to expand the analysis to other scales because viruses and vectors can be transported from all distances^61^ ^-^ ^63^.

This study provided evidence that ENSO influence over climatic variability has changed atmospheric conditions in the long term by decreasing rainfall and increasing temperatures. This can have influenced the dengue temporal patterns in the MRR through transient reduction of its main way of multiyear variability (3-4 years) to 2-3 years. Furthermore, when the epidemic coincided with El Niño years, it was widespread among municipalities and tightly synchronized. These findings are in line with previous studies that found evidence that ENSO is associated with changes in dengue epidemiology^24^ ^,^ ^39^ ^,^ ^42^ ^,^ ^44^, which is synchronized between different regions^64^. The results of this study provide an adequate basis for information to intensify combat and epidemiological control mechanisms when initial El Niño conditions are being established. Such results can also be used to support the control of Zika and Chikungunya virus, as they are all transmitted by the same vector, *Ae. Aegypti*.
